# Incidental Vascular Findings in Computed Tomography Performed in the Qualification for the TAVI Procedure

**DOI:** 10.3390/diagnostics12112773

**Published:** 2022-11-13

**Authors:** Paweł Gać, Aleksandra Grochulska, Rafał Poręba

**Affiliations:** 1 Centre for Diagnostic Imaging, 4th Military Hospital, Weigla 5, 50-981 Wroclaw, Poland; 2 Department of Population Health, Division of Environmental Health and Occupational Medicine, Wroclaw Medical University, Mikulicza-Radeckiego 7, 50-368 Wroclaw, Poland; 3 Department of Internal and Occupational Diseases, Hypertension and Clinical Oncology, Wroclaw Medical University, Borowska 213, 50-556 Wroclaw, Poland

**Keywords:** computed tomography, ectopic right coronary artery ostium, partial anomalous pulmonary venous drainage, persistent left superior vena cava, right aortic arch, TAVI

## Abstract

Transcatheter aortic valve implantation (TAVI) or transcatheter aortic valve replacement (TAVR) is now a very widespread treatment method for symptomatic and severe aortic stenosis as an alternative for patients at intermediate or high risk of surgery or contraindications to surgery. The key role of imaging examinations before TAVI is to assess the morphology of the aortic valve, the routes of surgical access, and non-cardiac and extravascular structures. The objective of this article is to present and discuss the importance of selected accidental vascular findings in computed tomography examinations of the heart and large vessels performed in the TAVI qualification procedure: persistent left superior vena cava (SVC) with absent right SVC, right aortic arch, ectopic right coronary artery ostium, and left superior pulmonary vein draining into left brachiocephalic vein.

Transcatheter aortic valve implantation (TAVI) or transcatheter aortic valve replacement (TAVR) is now a very widespread treatment method for symptomatic and severe aortic stenosis as an alternative to high-risk surgery.

According to the European ESC guidelines of 2021, TAVI is recommended in older patients (≥75 years) or in those who are high-risk (STSPROM/EuroSCORE II > 8%) or unsuitable for surgery—class I recommendations. Surgical aortic valve replacement (SAVR) is recommended in younger patients who are low-risk for surgery (<75 years and STS-PROM/EuroSCORE II < 4%), or in patients who are operable and unsuitable for transfemoral TAVI—class I recommendations. SAVR or TAVI are recommended for the remaining patients according to individual clinical, anatomical, and procedural characteristics—class I recommendations. Non-transfemoral TAVI may be considered in patients who are inoperable and unsuitable for transfemoral TAVI—class IIb recommendations [1].

In accordance with the above guidelines, the clinical, anatomical, and procedural factors that indicate TAVI as the preferred method include: higher surgical risk, older age, previous cardiac surgery (particularly, intact coronary artery bypass grafts at risk of injury during repeat sternotomy), severe frailty, TAVI is feasible via transfemoral approach, transfemoral access is challenging or impossible and SAVR is inadvisable, sequelae of chest radiation, porcelain aorta, high likelihood of severe prosthesis mismatch (AVA < 0.65 cm^2^/m^2^ BSA), and severe chest deformation or scoliosis [1]. Factors indicating a preference for SAVR over TAVI are lower surgical risk, younger age, active or suspected endocarditis, transfemoral access is challenging or impossible and SAVR is feasible, aortic annular dimensions are unsuitable for available TAVI devices, bicuspid aortic valve, valve morphology is unfavorable for TAVI (e.g., high risk of coronary obstruction due to low coronary ostia or heavy leaflet/LVOT calcification), thrombus in aorta or left ventricle, significant multivessel CAD requiring surgical revascularization, severe primary mitral valve disease, severe tricuspid valve disease, significant dilatation/aneurysm of the aortic root and/or ascending aorta, and septal hypertrophy requiring myectomy [1].

Initially, TAVI was considered a promising minimally invasive method for patients disqualified from SAVR. The results obtained from the comparative TAVI and SAVR studies have unequivocally proved that both types of treatment can be considered comparable in terms of mortality and postoperative morbidity. However, the development of technology along with the operator learning curve create prospects for improving the effects of TAVI treatment [2].

The key role of imaging examinations before TAVI is to assess the morphology of the aortic valve, the routes of surgical access, and non-cardiac and extravascular structures. Assessing the morphology of the aortic valve involves assessing the number of valve leaflets and the degree and location of its calcifications. A bicuspid aortic valve or valve with massive calcification predisposes to PVL (paravalvular leak), PPM (permanent pacemaker), TVE (transcatheter valve embolization), or annulus rupture. The assessment of calcifications in the LVOT (left ventricular outflow tract) is also important—massive calcifications in the LVOT also predispose to PVL (paravalvular leak), annulus rupture, iVSD (iatrogenic ventricular septal defect), and PPM (permanent pacemaker) [3]. It should be noted that one of the challenges in the assessment of calcification in computed tomography is the problem of blooming artifacts, due to which the accuracy of the assessment may be lower. De-blooming algorithms or manual extraction are potential solutions to increase the accuracy of refining the boundaries of calcifications [4,5].

Assessing surgical access routes involves assessing the width and height of the aortic bulb; assessing the width of the ascending aorta (measured about 4–5 cm above the level of the aortic annulus); assessing calcifications in the ascending aorta, in the iliac, femoral, and subclavian arteries; and detecting implanted bypass anastomoses and stents, aneurysms, and silent dissections of the vascular wall [3]. Performing multidetector computed tomography (MDCT) in the TAVI qualification procedure reduces the incidence of vascular complications related to the aorta (aortic/annular dissection or rupture, left ventricular rupture, pseudoaneurysm/aneurysm) or peripheral access (dissection, stenosis, rupture, fistula, pseudoaneurysm, hematoma). The incidence of complications related to unplanned surgical/intravascular interventions, and the incidences of visceral ischemia or neurological disorders are also lower [6].

Extra-cardiac and extravascular structures are assessed to identify changes that modify the risk factors of the procedure, i.e., proliferative processes [3]. Minamito-Muta et al. assessed the impact of cancer on the prognosis of patients with severe aortic stenosis, as well as the therapeutic strategy in the case of aortic valve stenosis with malignant neoplasm. They showed that, in patients with active cancer and severe aortic stenosis, the main reasons for choosing the SAVR/TAVI strategy were symptoms related to aortic valve stenosis, as well as the need for aortic valve replacement before non-cardiac surgery and very severe aortic stenosis. The most common reasons for choosing a conservative treatment strategy were an absence of symptoms, limited life expectancy due to diseases unrelated to aortic stenosis, and lack of patient consent to SAVR/TAVI [7].

The objective of this article is to present and discuss the importance of selected incidental vascular findings in MDCT examinations of the heart and large vessels performed in the TAVI qualification procedure.

All the presented studies were performed on a two-source, 384-slice CT (Somatom Force, Siemens Healthcare, Germany). The study protocol included a topogram, a native phase dedicated to the assessment of the aortic valve calcium score, premonitoring and monitoring at the level of the bifurcation of the trachea with image acquisition triggered by reaching a density of 100 HU in the ROI placed in the ascending aorta, an angiographic phase dedicated to the morphological evaluation of cardiac structures, and an angiographic phase dedicated to imaging all potential TAVI accesses. The acquisition of the native and angiographic phases for cardiac morphology ranged from tracheal bifurcation to the diaphragm, and the angiographic phase for imaging potential TAVI accesses ranged from the hyoid bone to the lesser femoral trochanter. An X-ray tube kilovolt of 100 kV was used, and the mAs values were variable. Using an automatic injector, 80 mL of an iodine non-ionic contrast agent (350 mgJ/mL) was administered into a vein of the ulnar fossa at an injection rate of 4.0 mL/s.

Incidental vascular finding No. 1: Persistent Left Superior Vena Cava with Absent Right Superior Vena Cava.

Image description: A 73-year-old patient with an implanted vascular port in the left subclavian region, the end of the port is visible in the left superior vena cava, with the simultaneous absence of the right superior vena cava.

The suspicion of venous confluence abnormalities was known earlier due to the course of the vascular port catheter on the control chest radiograph taken after vascular port implantation.

The axial CT scan (Figure 1) shows the left superior vena cava with the catheter in its lumen (red arrow), while the right superior vena cava is not visible.

The computed tomography image in the MIP reconstruction, in the coronal view (Figure 2), shows the venous confluence on the right side as the right internal jugular vein connecting with the right subclavian vein into the right brachiocephalic vein (red star), the venous confluence on the left side as the left internal jugular vein connecting with the subclavian vein the left brachiocephalic vein (blue star), the atypical course of the right brachiocephalic vein crossing the proximal branches of the aortic arch from the front (yellow star), and its connection with the left brachiocephalic vein into the left superior vena cava (green star).

Discussion: Persistent left superior vena cava is a developmental anomaly of venous circulation occurring in about 0.5% of the general population, of which about 0.3% are healthy people and 4.3% are people with congenital heart defects. Even more rare are cases with persistent left SVC in the absence of right SVC, and they account for about 0.09% to 0.13% of patients with congenital heart disease. Defects that coexist with this anomaly include situs inversus or situs solitus [8].

An absent right SVC can lead to an inability to insert central venous canula, pulmonary artery catheter, and transvenous pacing leads through the right internal jugular or subclavian vein. In addition, a persistent left SVC may lead to several clinical issues, which include the following:

- Difficulty inserting central venous cannula, pulmonary artery catheter, and transvenous pacing leads through the left internal jugular/subclavian vein. The dilated coronary sinus (CS), which is thin-walled and stretched, predisposes it to perforation, tamponade, various arrhythmias, and cardiac arrest.
- Persistent left SVC may require separate cannulation during right-sided open-heart surgical procedures, either directly or through CS.
- Difficulty in performing cavo-pulmonary anastomoses.
- Inability to deliver retrograde cardioplegia during open-heart surgery and port-access cardiac surgery because of the large size of CS.
- Rhythm abnormalities such as ectopic atrial rhythm, wandering pacemaker, first-degree atrioventricular block, tachyarrhythmia, and complete heart block because of the stretching or fragmentation of the atrioventricular node and His bundle secondary to the dilatation of CS. Sinoatrial nodal abnormalities have been detected in patients with absent right SVC, predisposing the patient to sick sinus syndrome.
- Rarely, enlarged CS can impinge on the vestibule of the mitral valve that may produce obstruction to left ventricular inflow.
- An orthotopic heart transplant may require the recipient’s heart to be excised along the atrioventricular grove, preserving the persistent left superior vena cava and CS [9].

In view of the above-mentioned anticipated intraoperative implications, it is advisable to investigate a dilated CS more than 1 cm in diameter thoroughly. The enlargement of the CS may indicate right atrial hypertension, an anomalous systemic venous channel, fistulous communication with coronary arteries, and total or partial anomalous pulmonary venous drainage.

As discussed above, persistent left superior vena cava with absent right superior vena cava is an anomaly of importance, especially in surgical procedures. For this reason, its diagnosis should be considered when choosing a method of aortic valve replacement—it seems that TAVI is safer than SAVR.

Incidental vascular finding No. 2: Right Aortic Arch.

Image description: An 80-year-old patient with a right type II aortic arch and an aberrant left subclavian artery.

The computed tomography image in the MIP reconstruction, in the axial view (Figure 3), showed a right aortic arch (red arrow).

The computed tomography images in the VRT reconstruction, in the coronal anterior and posterior view (Figure 4A,B), showed the right aortic arch and its branches: aberrant left subclavian artery (green line), right common carotid artery (pink line), right subclavian artery (purple line), and left common carotid artery (blue line). A previously implanted aortic-coronary bypass was also visualized (black lines).

Discussion: A right aortic arch is a developmental variant of the aortic arch in which the aortic arch points to the right side of the trachea. It can come in various configurations. Type I is a right aortic arch with a mirror image of its branches (it accounts for about 59% of cases). It often coexists with cyanotic defects such as: tetralogy of Fallot, aortic trunk, translation of great arterial trunks, and trivalve atresia. Type II is a right aortic arch with an aberrant left subclavian artery. It accounts for about 39.5% of right aortic arch cases. It may coexist with Kommerell diverticulum. It rarely coexists with other cardiovascular defects. Type III is a right aortic arch with an isolated left subclavian artery [10]. It is the rarest type; it accounts for about 0.8% of cases. In planning TAVI, the determination of anatomical conditions is of key importance. With a right aortic arch and aberrant left subclavian artery, transfemoral access will constitute the safest and most sensible approach [11]. In patients with a right-sided aortic arch, devices to protect against cephalic embolism, such as the Sentinel CPS device (Claret Medical Inc, USA) or Embrella (Edwards Lifesciences, USA), are usually not applicable during the TAVI procedure [6].

Incidental vascular finding No. 3: Ectopic Right Coronary Artery Ostium.

Image description: A 77-year-old patient, in the process of qualification for TAVI. The examination showed a potentially malignant anomaly of the ostium of the right coronary artery (RCA) from the left sinus of Valsalva. RCA takes off from the left sinus of Valsalva, and runs between the aorta and the pulmonary trunk. The left coronary artery and the left circumferential artery were normal.

The computed tomography image in the MIP reconstruction, in the axial view (Figure 5), showed an ostium of the right coronary artery from the left sinus of Valsalva (red arrow).

In the computed tomography image in the VRT reconstruction, in the anterolateral view (Figure 6), the right coronary artery (green line) was visualized with the ostium from the left sinus of Valsalva, and the course of the proximal segment between the pulmonary trunk (blue star) and the aorta (red star).

Discussion: Congenital anomalies of the coronary arteries are quite rare, with a frequency of 0.17–1.2% in the population. In most patients, the first symptom of their presence may be sudden death; others are angina, shortness of breath, and fainting. Moreover, it is known that the inlet area of the coronary artery is prone to atherosclerosis, in which the hemodynamic metric, in particular, wall shear stress, may play an important role. Therefore, the meaning of the anatomic variations on the risk of atherosclerosis in the proximal segment of coronary arteries should be further explored [12].

It is important that the clinician should be aware of this vascular anomaly prior to the procedure of aortic valve implantation and be able to appropriately modify his/her choices regarding the method of implantation as well as the type of valve to minimize the risk of compression of the anomalous coronary artery by the expanding prosthetic valve [13]. In patients requiring aortic valve replacement with a coexisting potentially malignant coronary anomaly who qualify for TAVI, potential remedies (e.g., prophylactic wire placement in the artery to facilitate stent placement) should be considered. In this group of patients, it is also necessary to consider possible surgical treatment: SAVR with simultaneous coronary unroofing, coronary reimplantation, or a coronary artery bypass graft [14].

Incidental vascular finding No. 4: Left Superior Pulmonary Vein Draining into Left Brachiocephalic Vein.

Image description: An 80-year-old patient—an examination before planned TAVI to determine vascular access and vascular anatomy. Abnormal drainage of the left superior pulmonary vein into the left brachiocephalic vein was shown.

The computed tomography image in the MIP reconstruction, in the oblique view (Figure 7), showed an unusually upward left superior pulmonary vein (green arrow).

The computed tomography images in the VRT reconstruction, in the coronal anterior view (Figure 8), showed abnormal drainage of the left superior pulmonary vein (purple star) into the left brachiocephalic vein (red star).

Discussion: The left superior pulmonary vein draining into the left brachiocephalic vein is a variation of partial anomalous pulmonary venous drainage (PAPVD). In their case, extrapulmonary left–right leakage is observed. On the left, the superior pulmonary vein drains into an additional vertically running vessel at the aortic arch, which corresponds to a fragment of the persistent superior vena cava that drains into the left brachiocephalic vein. Consequently, there is no adequate vein drainage to the left atrium [15]. In patients qualified for aortic valve replacement, in the case of a diagnosis of PAPVD, switching to surgery should be considered, e.g., simultaneous aortic valve replacement and venous return correction [16]. In diagnostic procedures, it is necessary to determine the volume of the shunt and to evaluate the right heart function using magnetic resonance imaging or cardiac catheterization [17].

## Figures and Tables

**Figure 1:**
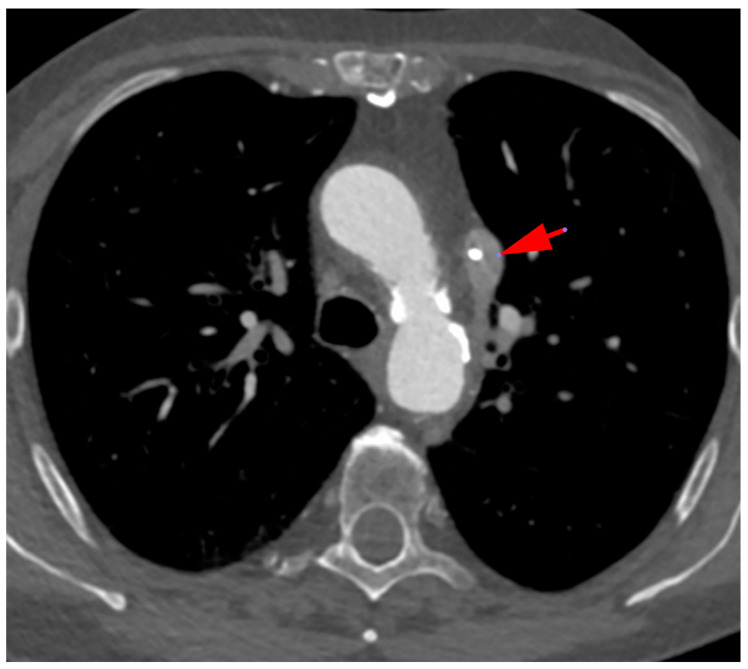
Persistent left superior vena cava (SVC) with absent right SVC.

**Figure 2:**
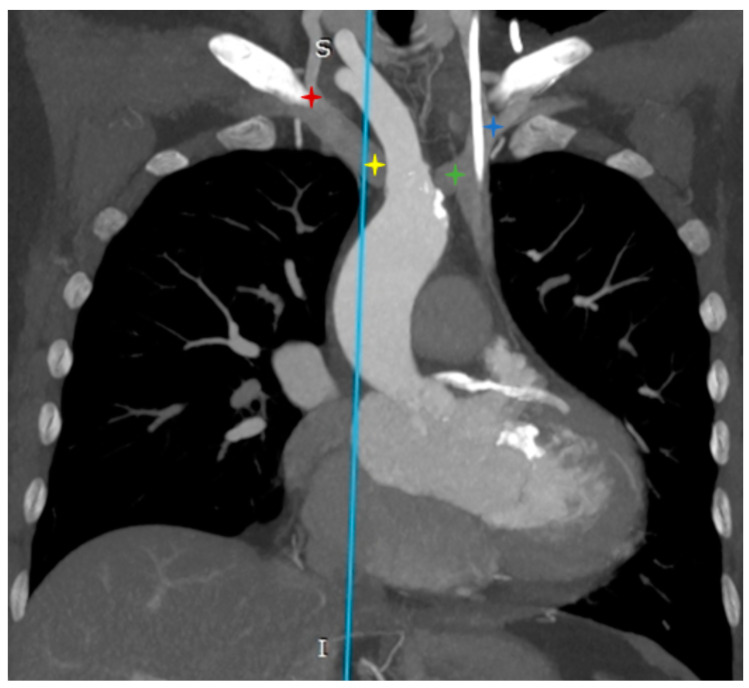
Confluence of the brachiocephalic veins to the left superior vena cava.

**Figure 3:**
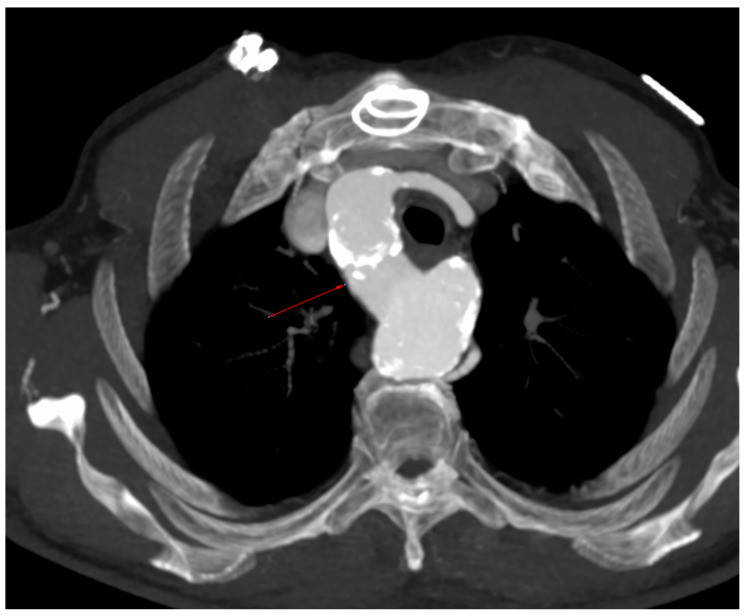
Right aortic arch.

**Figure 4:**
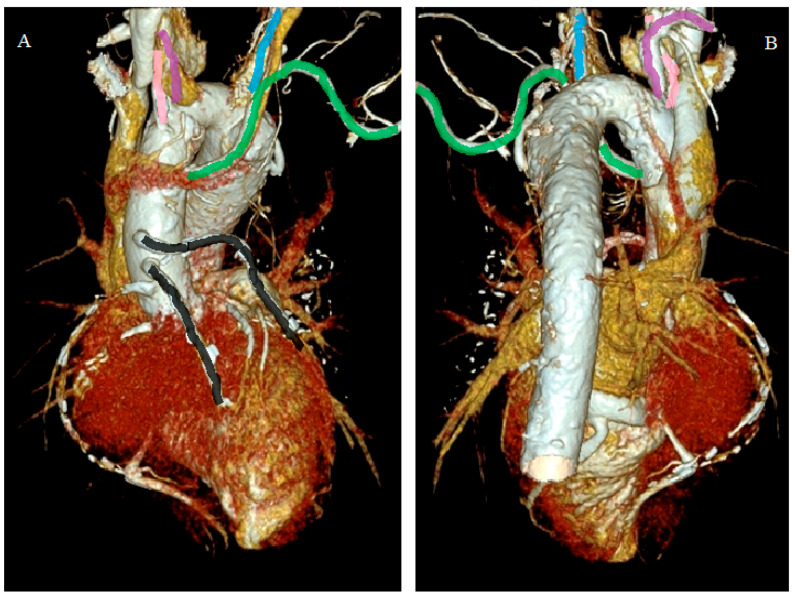
The right aortic arch and its branches. (**A**) Coronal anterior view. (**B**) Coronal posterior view.

**Figure 5:**
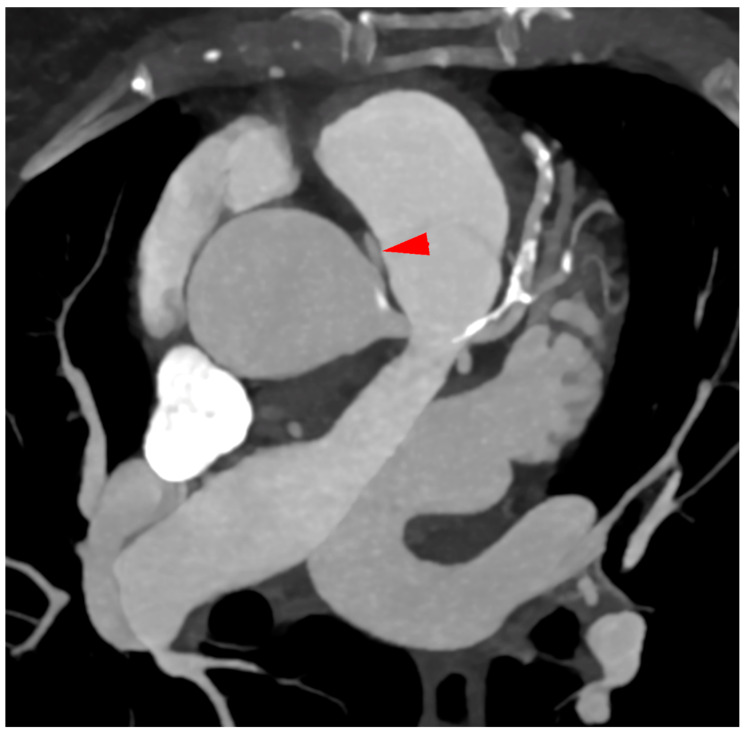
The ostium of the right coronary artery.

**Figure 6:**
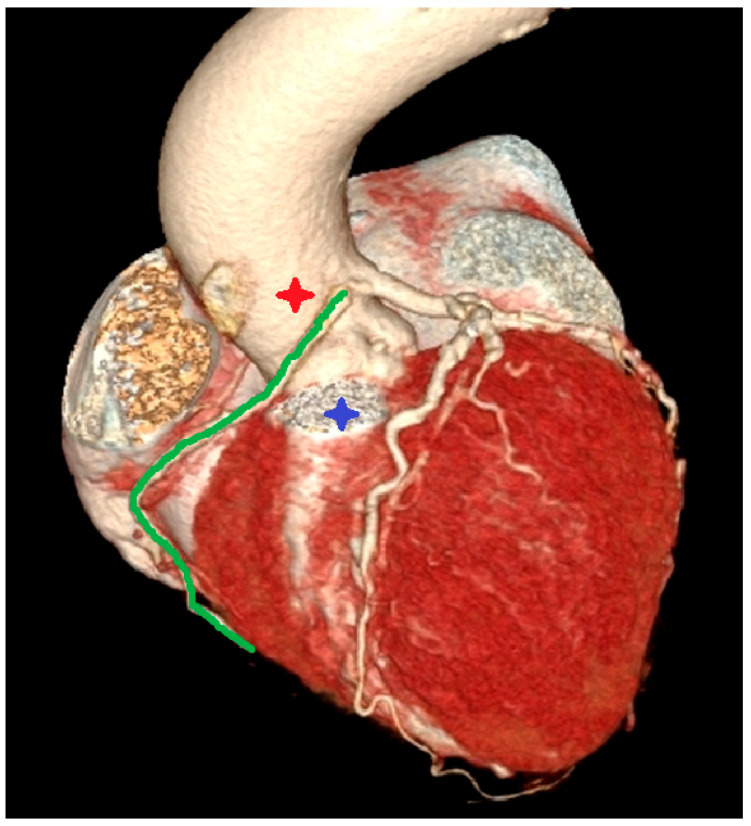
Interarterial course of the right coronary artery.

**Figure 7:**
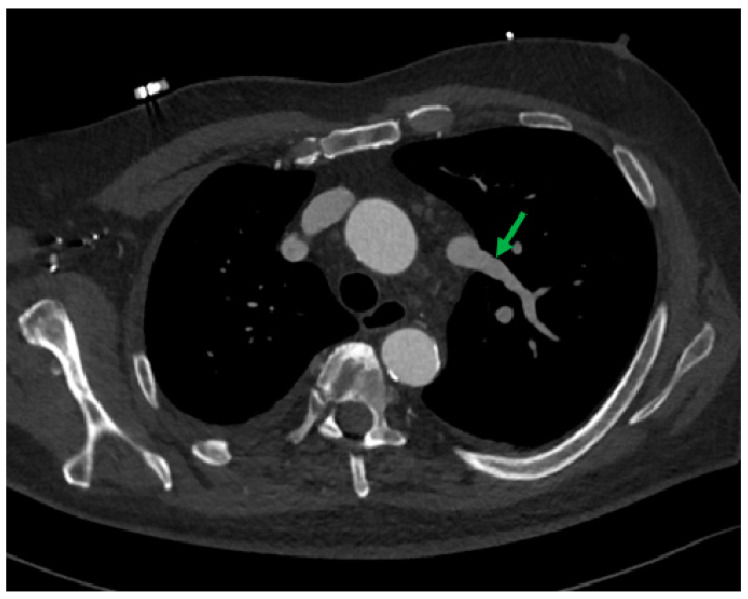
Unusually upward left superior pulmonary vein.

**Figure 8:**
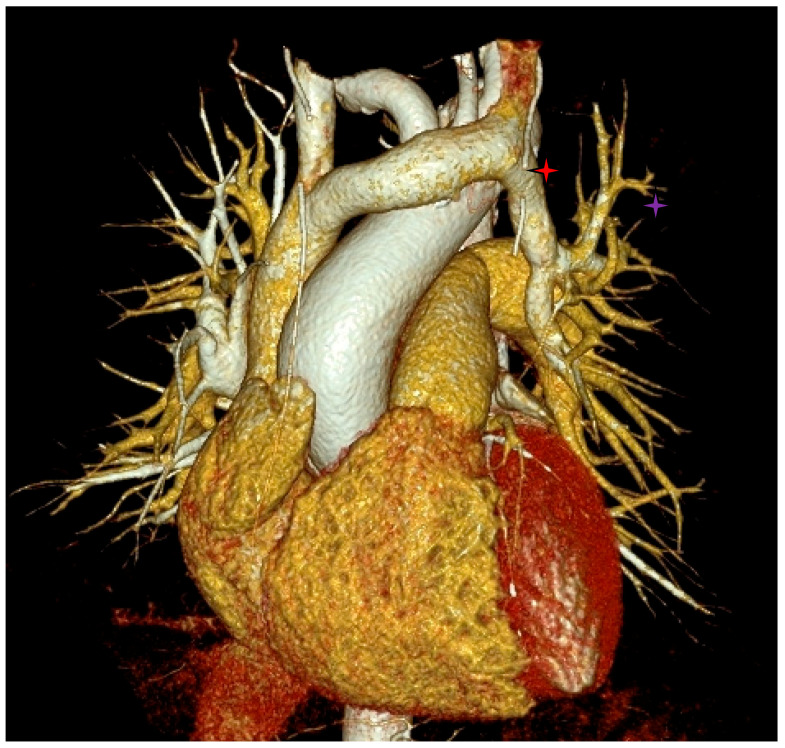
Left superior pulmonary vein draining into left brachiocephalic vein.
